# Splenectomy with Portoazygous Disconnection for Correction of Systemic Hemodynamic Disorders in Hepatic Cirrhosis Patients with Portal Hypertension: A Prospective Single-Center Cohort Study

**DOI:** 10.1155/2020/8893119

**Published:** 2020-12-21

**Authors:** Dao-Bing Zeng, Liang Di, Qing-Liang Guo, Jing Ding, Xiao-Fei Zhao, Shi-Chun Lu

**Affiliations:** ^1^General Surgery Department, Beijing Youan Hospital, Capital Medical University, Beijing 100069, China; ^2^Institute and Hospital of Hepatobiliary Surgery of Chinese PLA, Chinese PLA Medical School, Chinese PLA General Hospital, Beijing 100853, China

## Abstract

**Objective:**

To investigate the effect of splenectomy for correction of systemic hemodynamic disorders in hepatic cirrhosis patients with portal hypertension.

**Methods:**

Hepatic cirrhosis patients with portal hypertension were enrolled from April 2015 to July 2018. Systemic hemodynamic parameters (heart rate, mean arterial pressure (MAP), cardiac output, and total peripheral vascular resistance (TPR)) were prospectively measured at baseline and 1 week, 1, 3, and 6 months, and 1, 2, and 3 years postoperatively. Paired analysis was conducted.

**Results:**

Sixty-nine patients were eligible, and 55 (79.7%) cases had a history of upper gastrointestinal bleeding. Child–Pugh classification was grade A in 41 (59.4%) cases, grade B in 26 (37.7%) cases, and grade C in 2 (2.9%) cases. The heart rate was significantly higher at 1 week postoperatively versus the baseline (*P* < 0.001). Meanwhile, the heart rate was significantly lower from 3 months to 2 years postoperatively versus the baseline (*P* < 0.05). The MAP was significantly higher at 6 months to 2 years postoperatively versus the baseline (*P* < 0.05). At 1 month postoperatively and 6 months to 2 years, the cardiac output was significantly lower versus the baseline (*P* < 0.05). At 1 month postoperatively and 6 months to 2 years, the TPR was significantly higher versus the baseline (*P* < 0.05).

**Conclusion:**

Splenectomy corrects systemic hemodynamic disorder in hepatic cirrhosis patients with portal hypertension, and the effect is rapid and durable.

## 1. Introduction

Decompensated hepatic cirrhosis represents the end stage of chronic liver disease and is characterized by the development of clinically evident complications of portal hypertension and liver insufficiency with pre-existing fibrosis. The main manifestations of portal hypertension include esophagogastric varices with or without upper gastrointestinal tract bleeding, splenomegaly and hypersplenism, and ascites. Hemodynamic disorders of cirrhotic portal hypertension include systemic hemodynamic disorders and visceral hemodynamic disorders. Systemic hemodynamic disorders include increased cardiac output (CO) and heart rate (HR) and reduced systemic arterial blood pressure and peripheral vascular resistance [1]. Hemodynamic changes such as portal hypertension and hyperdynamic circulation are major causes of morbidity and mortality in patients with liver cirrhosis [2–4].

At present, only drug therapy [5] and liver transplantation [6] can effectively correct systemic hemodynamic disorders in patients with cirrhosis and portal hypertension. Splenectomy for correction of hemodynamic disorders in patients with liver cirrhosis focuses only on visceral hemodynamics [7–9] while there has been no report on the correction of systemic hemodynamic disorders. In the current prospective study, we investigated the effect of splenectomy on hemodynamic parameters in hepatic cirrhosis patients with portal hypertension.

## 2. Patients and Methods

### 2.1. Patients

This prospective single-center cohort study carried out between April 1, 2015, and August 15, 2018, enrolled liver cirrhosis patients with portal hypertension who underwent splenectomy with portoazygous disconnection at Beijing Youan Hospital, Capital Medical University, Beijing, China. Cirrhosis was confirmed by either pathological or radiological evidence of liver nodularity or esophageal varices with a combination of clinical presentations and laboratory results indicating portal hypertension. The inclusion criteria were as follows: (1) patients aged between 20 and 65 years; (2) clinically or pathologically confirmed cirrhosis and portal hypertension (including viral hepatitis, alcoholic hepatitis, and autoimmune hepatitis); (3) the diameter of the splenic artery >5.19 mm or the ratio of the splenic artery diameter to the proper hepatic artery diameter >1.4; (4) preoperative evaluation showed stable vital signs and Child–Pugh grade *A*, *B*, and *C*; and (5) patients who could tolerate abdominal surgery under general anesthesia. Major exclusion criteria were as follows: (1) idiopathic portal hypertension; (2) Budd–Chiari syndrome; (3) poor compliance; preoperative evaluation showed vital sign instability and the need to use vasoactive drugs to maintain blood pressure; overt hepatic encephalopathy [10]; coagulation dysfunction; and hepatocellular carcinoma; (4) a history of hypertension; and (5) use of *β*-blockers or other medications that could affect systemic hemodynamics.

The study protocol was approved by the Ethics Committee of Capital Medical University Beijing Youan Hospital (Approval No. [2018]006). All patients were informed of the experimental nature of the surgery due to lack of current guidelines and provided written informed consent. The study was conducted in accordance with the Declaration of Helsinki.

### 2.2. Surgical Procedures

All patients underwent laparotomy. All surgical procedures were performed by the same team of surgeons with more than 10 years of experience in hepatobiliary surgery. Patients with a history of upper gastrointestinal bleeding received splenectomy with portoazygous disconnection as described [11]. Patients with no previous history of upper gastrointestinal hemorrhage underwent portal azygos disconnection according to portal vein pressure after splenectomy [12]. Briefly, a subcostal incision was made in the left upper abdomen. The right gastroepiploic vein was dissected and cannulated, and the portal vein pressure was measured using the column of water method. Then, the gastrocolic ligament was dissociated, and the splenic artery was dissected in the superior border of the pancreas and then ligated using No. 7 silk suture. The spleen was massaged, and the ligatures around the spleen were transected. The spleen was dissociated, and the pedicle of the spleen was transected at the hilum. After the portal vein pressure was measured, the varicose veins in the greater and lesser curvature of the stomach and the lower esophagus (6–10 cm) were transected.

### 2.3. Patient Evaluation

Eligible patients were followed up once at one, three, and six months postoperatively and once every three to six months postoperatively in the clinic. Each visit included physical examination, blood tests, and abdominal ultrasound. Follow-up data were gathered until 36 months from baseline, and the last day of follow-up visit was August 15, 2018. Patient demographics, complications of cirrhosis, routine laboratory tests (standard liver and renal function tests), and blood chemistries including white blood cell (WBC) counts and platelet count were collected. The model for end-stage liver disease (MELD) and Child–Turcotte–Pugh (CTP) scores were calculated. An Ultrasound CO monitor (USCOM) was used to measure CO at baseline, 1 week, 1, 3, and 6 months, and 1, 2, and 3 years postoperatively. CO data were censored if the ejection velocity did not fall between 1.2 m/s and 1.8 m/s as instructed by the manufacturer (Uscom, Sydney, Australia). HR and blood pressure were measured simultaneously, and calculated by the following formulae [13]:

Mean arterial pressure (MAP) = (pulse pressure/3) + diastolic blood pressure and total peripheral resistance (TPR) = MAP^*∗*^80/CO.

In addition, the incidence of variceal bleeding was recorded.

### 2.4. Statistical Analysis

Quantitative variables were expressed by mean ± standard deviation ($\bar{x}$ ± *s*), and qualitative data were presented as frequency and percentages. Data were analyzed using SPSS 22.0 (SPSS Inc., Chicago, IL, USA). A paired *t*-test was used to compare postoperative results with the baseline data. *P* < 0.05 was statistically significant.

## 3. Results

### 3.1. Demographic and Baseline Characteristics of the Study Population

Totally 69 patients were eligible for the study, including 39 (56.5%) males and 30 (43.5%) females. Their mean age was 47.8 ± 9.7 years (range 25–65). Hepatic cirrhosis was associated with hepatitis *B* in 39 (56.5%) cases, alcoholic abuse in 4 (2.9%) cases, both hepatitis B and alcoholic abuse in 5 (7.3%) cases, and autoimmune cirrhosis in 8 (11.6%) cases. Fifty-five (79.7%) cases had a history of upper gastrointestinal bleeding. Child–Pugh classification of liver function was grade *A* in 41 (59.4%) cases, grade *B* in 26 (37.7%) cases, and grade *C* in 2 (2.9%) cases. The median MELD score was 5 (range −3, 14). The demographic and baseline characteristics of the study population are shown in Table 1.

### 3.2. HR and MAP

The median follow-up duration was 29 months (range: 1 month to 38 months). Seven cases were lost to follow-up, and 2 cases withdrew from the study (hepatocellular carcinoma was diagnosed in 1 case 1 year postoperatively, and 1 case developed recurrent hepatic encephalopathy after operation). One case died of intracranial hemorrhage due to trauma at 10 months postoperatively. The HR was significantly higher at 1 week postoperatively versus the baseline (*P* < 0.001). Meanwhile, the HR was significantly lower from 3 months to 2 years postoperatively versus the baseline (*P* < 0.05) (Table 2).

Furthermore, there was no statistically significant difference in MAP at 1 week to 3 months postoperatively and the baseline (*P* > 0.05) (Table 3). Meanwhile, the MAP was significantly higher at 6 months to 2 years postoperatively and the baseline (*P* < 0.05), with a mean increase of 6.23 ± 3.2 mmHg at 2 years postoperatively. In addition, though no statistically significant difference in MAP was observed at 3 years postoperatively and the baseline (*P*=0.068), an increase of 7.63 ± 2.29 mmHg over the baseline was observed at 3 years postoperatively.

### 3.3. CO and TPR

There was no statistically significant difference in CO at 1 week postoperatively and the baseline (*P* > 0.05) (Table 4). At 1 month postoperatively and 6 months to 2 years, the CO was significantly lower compared with the baseline (*P* < 0.05). No statistically significant difference in CO was observed at 3 months and 3 years postoperatively versus the baseline (*P* > 0.05).

Moreover, there was no statistically significant difference in TPR at 1 week postoperatively and the baseline (*P* > 0.05) (Table 5). At 1 month postoperatively and 6 months to 2 years, the TPR was significantly higher compared with the baseline (*P* < 0.05). No statistically significant difference in TPR was observed at 3 months and 3 years postoperatively versus the baseline (*P* > 0.05).

### 3.4. Variceal Bleeding, Thrombosis, and Infections

The portal vein pressure was significantly lower than that of the baseline (25.76 ± 4.70 cmH_2_O *vs.* baseline: 34.13 ± 6.80 cm H_2_O, *P* < 0.001). At the end of follow-up, upper gastrointestinal bleeding was reported in 5 (7.25%) cases. Two cases of upper gastrointestinal bleeding occurred within one month of surgery, which was stopped by medication and balloon tamponade and did not recur at the final follow-up. The remaining three cases of gastrointestinal bleeding occurred within one year of surgery, which mainly manifested as melena and was controlled by medication. No gastrointestinal bleeding recurred at the 2- and 3-year follow-up visit. All patients were alive at the last follow-up visit (August 15, 2018).

In addition, 32 (46.4%) patients developed portal vein thrombosis, including 6 (8.7%) Yerdel grade I or II cases in the superior mesenteric vein. Three (4.4%) patients had lung infection, which was cured by antibiotics therapy. No peritoneal or systemic infection occurred.

## 4. Discussion

In 1953, Kowalski and Abelmann first reported an increase in CO in patients with liver cirrhosis [14], which was subsequently confirmed by numerous studies [15–19]. The increase of CO is caused by increased stroke volume and HR. However, not all patients have an increase in CO; about 30% to 70% of hepatic cirrhosis patients have increased CO. The mean CO of cirrhosis patients is higher than that of controls (7.14 *vs.* 5.66 L/min; *P* < 0.05) [20]. Our study showed that CO underwent a significant gradual decline over time after splenectomy (6.91–6.24 L/min *vs.* 7 L/min at baseline). Although liver transplantation can completely treat hyperdynamic circulation in cirrhotic portal hypertension, Henderson et al. demonstrated that high CO persisted until 2 years after operation [21]. In our study, we also found significant difference in CO until 2 years postoperatively but observed no difference at 3 years postoperatively versus the baseline. These findings indicate that the effect of splenectomy on CO extends beyond the immediate postoperative period.

Numerous studies have confirmed that the HR of patients with cirrhosis is significantly faster than that of the control group [20]. Møller et al. [22] showed that intravenous injection of 2 mg terlipressin can reduce CO and HR (18% and 11%, respectively). However, the action of terlipressin lasted no more than 4 hours at the longest [23]. Terlipressin also has side effects which include moderate abdominal pain, arterial hypertension, hyponatremia, and severe cardiovascular and ischemic diseases in about 15% of patients, limiting its clinical application. Our study revealed that the baseline HR of the patients varied between 73.50 and 75.00 bpm, while it decreased significantly and varied between 64.24 bpm and 69.85 bpm from 3 months to 3 years after splenectomy. The findings demonstrate that splenectomy has a significant and durable effect on reducing HR in patients with liver cirrhosis.

Arterial blood pressure is a reliable index of circulatory function in patients with liver cirrhosis [24]. Systolic and diastolic blood pressure and MAP in patients with cirrhosis gradually decreased from Child–Pugh *A* to *C* (*P* < 0.001) [19]. Arterial blood pressure was negatively correlated with CO in patients with cirrhosis (*r* = −0.26, *P* < 0.01) [20]. Consistently, our study showed that CO decreased gradually after splenectomy, while MAP increased gradually. From 6 months to 2 years postoperatively, MAP was significantly higher than the baseline. The MAP at 3 years postoperatively was larger than the baseline, but there was no statistical difference, which may be related to the small sample size. We speculate that physiopathological changes occur as a result of hemodynamics corrections (reduction in HR and CO and rise in MAP and TRP) after splenectomy and portoazygous disconnection as follows: splenectomy markedly reduces portal vein pressure by 30%–50% and decreases blood flow *via* the portal vein by approximately 30% [7]. It also increases blood flow through the hepatic artery and reduces the hepatic artery resistance index and improves renal function. As a result, blood volume is restored near normal levels.

The arterial compliance of patients with cirrhosis was significantly higher than that of the control group (1.39 *vs.* 1.04 mL/mmHg; *P* < 0.01), and TPR decreased significantly as Child–Pugh *A* decreased to *C* (*P* < 0.001) [20]. Although terlipressin increases blood pressure and systemic vascular resistance (26% and 61%, respectively), its efficacy is limited, and it also has many side effects [22]. Studies have shown that MAP is closely associated with hepatic function and survival of patients. Systolic and diastolic blood pressure and MAP progressively decline in patients as hepatic cirrhosis patients progress from Child–Pugh *A* to Child–Pugh *C* [20]. The 1-year survival rate of patients with hepatic cirrhosis with a MAP <82 mm/Hg was 40% while that of those with MAP>82 mm/Hg was 70% [25]. A study has shown that a history of hypertension is a protective factor against liver-related death and clinical decompensation as cirrhotic patients with hypertension do not have systemic peripheral vascular dilation because they are hyperdynamic with a low central volume. [24] Other studies have confirmed that pentoxifylline can increase systemic vascular resistance and reduce cardiac index in patients with alcoholic cirrhosis but has no effect on survival rate and portal pressure [26]. Our study showed that TPR increased from 1 month postoperatively and gradually increased over time up to 2 years postoperatively. There was no statistical difference in TPR at 3 years postoperatively and the baseline, which may be due to the small sample size. These findings show that splenectomy can not only increase TPR but also reduce portal vein pressure [7, 12] and improve survival rate [12].

In conclusion, splenectomy can not only correct systemic hemodynamic disorder in cirrhosis patients with portal hypertension but also has a rapid and lasting effect. It is the only effective treatment besides liver transplantation.

## 5. Conclusions

Splenectomy can not only correct systemic hemodynamic disorder in cirrhosis patients with portal hypertension but also has a rapid and durable effect. It is the only effective treatment besides liver transplantation.

## Figures and Tables

**Table 1:** Patient demographic and baseline characteristics.

Variables	All
No.	69

Age, years	
Mean (SD)	47.8 (9.7)
Range	25–65
Male gender, *n* (%)	39 (56.5)

Causes of cirrhosis, *n* (%)	
Hepatitis B	39 (56.5)
Hepatitis C	7 (10.1)
Alcoholic cirrhosis	4 (5.8)
Hepatitis B and alcoholic cirrhosis	5 (7.3)
Autoimmune	8 (11.6)
Idiopathic	1 (1.5)
Unknown	5 (7.3)
Previous variceal bleeding, *n* (%)	55 (79.7)
Hepatic encephalopathy, *n* (%)	2 (2.9)
Ascites, *n* (%)	40 (58.0)
Total bilirubin, *μ*mol/L, mean (SD)	21.52 (11.91)
Mean ALT (SD), U/L	21.76 (10.20)
Mean AST (SD), U/L	28.32 (13.87)
Albumin, g/L, mean (SD)	36.47 (4.25)
Mean creatine (SD), *μ*mol/L	63.28 (16.38)
Mean Na (SD), mmol/L	140.38 (3.03)
Mean INR (SD)	1.23 (0.15)
Mean CTP (SD)	6.29 (1.14)
Mean WBC (SD), ×10^9^/L	2.06 (1.08)
Lymphocytes, *n* (%)	28.38 ± 9.14
Neutrophils, *n* (%)	60.87 ± 10.23
Mean platelet (SD), ×10^9^/L	53.33 (35.49)

Child–Pugh classification	
Grade A	41 (59.4)
Grade B	26 (37.7)
Grade C	2 (2.9)

MELD	
Median (range)	5 (−3, 14)
Mean (SD)	5.06 (4.17)

ALT: alanine aminotransferase; AST: aspartate aminotransferase; CRP: C-reactive protein; CTP: Child–Turcotte–Pugh; INR: International Normalized Ratio; MELD: Model of End-Stage Liver Disease; SD: standard deviation; WBC: white blood cells. ALT male: 9–50 U/L, female: 7–40 U/L; AST male: 15–40 U/L, female: 13–35 U/L; total bilirubin 5–21 *μ*mol/L; albumin 40–55 g/L; creatinine 57–97 *μ*mol/L; Na 137–147 mmol/L; INR 0.8–1.2; WBC 3.5–9.5 × 10^9^/L; lymphocytes (%) 20–50%; neutrophils, *n* (%) 40–75%; platelet 125–350 × 10^9^/L.

**Table 2:** Comparison of heart rates at baseline and postoperatively in liver cirrhosis patients.

Time from surgery	No.	Heart rate (bpm)	*P* value	
		Baseline	After operation	
1 week	59	73.25 ± 9.11	78.07 ± 11.40	0.001
1 month	55	74.24 ± 10.35	74.14 ± 10.61	0.949
3 months	47	74.24 ± 10.75	69.75 ± 9.04	0.013
6 months	45	75.00 ± 10.77	69.85 ± 9.27	0.005
1 year	44	74.21 ± 10.57	67.92 ± 8.45	<0.001
2 years	17	74.63 ± 12.05	64.24 ± 8.82	0.002
3 years	10	73.50 ± 12.65	66.50 ± 8.30	0.112

**Table 3:** Comparison of mean arterial pressure at baseline and postoperatively in liver cirrhosis patients.

Time from surgery	No.	MAP (mmHg)		*P* value	
		Baseline	After operation	Mean change (postoperative baseline)	
1 week	59	82.83 ± 8.98	83.96 ± 8.83	1.13 ± 0.15	0.287
1 month	55	83.22 ± 8.71	85.22 ± 9.25	2.00 ± 0.54	0.097
3 months	47	83.09 ± 8.92	84.61 ± 9.20	1.52 ± 0.28	0.266
6 months	45	83.25 ± 9.30	88.89 ± 11.45	5.64 ± 2.15	0.001
1 year	44	83.62 ± 9.64	88.55 ± 12.91	4.93 ± 3.27	0.004
2 years	17	82.12 ± 11.16	88.35 ± 14.36	6.23 ± 3.2	0.035
3 years	10	84.47 ± 13.71	92.10 ± 11.42	7.63 ± 2.29	0.068

**Table 4:** Comparison of cardiac output (CO) at baseline and postoperatively in liver cirrhosis patients.

Time from surgery	No.	CO (L/min)	*P* value	
		Baseline	After operation	
1 week	38	7.37 ± 1.60	7.43 ± 1.29	0.797
1 month	31	7.62 ± 1.42	6.91 ± 1.19	0.005
3 months	26	7.24 ± 1.68	6.96 ± 1.24	0.298
6 months	25	7.61 ± 1.54	6.81 ± 1.79	0.021
1 year	26	7.05 ± 1.62	6.46 ± 1.38	0.033
2 years	8	7.32 ± 1.70	6.24 ± 0.95	0.048
3 years	5	6.79 ± 1.60	6.24 ± 0.84	0.474

**Table 5:** Comparison of total peripheral resistance (TPR) at baseline and postoperatively in liver cirrhosis patients.

Time from surgery	No.	TPR (dyne/s/cm^−5^)	*P* value	
		Baseline	After operation	
1 week	38	928.78 ± 233.51	908.79 ± 153.85	0.522
1 month	31	890.26 ± 199.53	999.25 ± 150.36	0.006
3 months	26	965.43 ± 260.62	996.89 ± 193.79	0.510
6 months	25	897.74 ± 224.71	1065.25 ± 238.98	0.002
1 year	26	992.41 ± 267.65	1085.20 ± 212.81	0.045
2 years	8	890.87 ± 222.48	1109.77 ± 201.62	0.014
3 years	5	962.60 ± 225.83	1147.70 ± 132.44	0.109

## Data Availability

The data used to support this study can be found in the text.
